# ﻿Taxonomy and molecular phylogeny of *Trametopsis* (Polyporales, Basidiomycota) with descriptions of two new species

**DOI:** 10.3897/mycokeys.90.84717

**Published:** 2022-05-31

**Authors:** Shun Liu, Yi-Fei Sun, Yan Wang, Tai-Min Xu, Chang-Ge Song, Yuan-Yuan Chen, Bao-Kai Cui

**Affiliations:** 1 Institute of Microbiology, School of Ecology and Nature Conservation, Beijing Forestry University, Beijing 100083, China Beijing Forestry University Beijing China; 2 College of Forestry, Henan Agricultural University, Zhengzhou, Henan 450002, China Henan Agricultural University Zhengzhou China

**Keywords:** Irpicaceae, macrofungi, multi-gene phylogeny, new species, white-rot fungi

## Abstract

*Trametopsis* is a worldwide genus belonging to Irpicaceae in the phlebioid clade, which can cause a white decay of wood. Previously, only three species were ascribed to the genus. In this study, we performed a morphological and phylogenetic study of *Trametopsis*. Molecular phylogenetic analyses of multiple loci included the internal transcribed spacer (ITS) regions, the large subunit nuclear ribosomal RNA gene (nLSU), the largest subunit of RNA polymerase II (RPB1), the second largest subunit of RNA polymerase II (RPB2) and the translation elongation factor 1-α gene (TEF1). Phylogenetic trees were inferred from the combined datasets of ITS+nLSU sequences and ITS+nLSU+RPB1+RPB2+TEF1 sequences by using maximum parsimony, maximum likelihood and Bayesian inference analyses. Combined with molecular data, morphological characters and ecological traits, two new species of *Trametopsis* are discovered. *Trametopsisabieticola* is characterised by its pileate, solitary or imbricate basidiomata, buff to buff-yellow pileal surface when fresh, becoming pinkish buff to clay-buff when dry, cream to buff pore surface when fresh, becoming pinkish buff to greyish brown upon drying, round to angular and large pores (0.5–1 per mm), cylindrical basidiospores (5.8–7.2 × 1.9–2.6 μm), distributed in the high altitude of mountains and grows on *Abies* sp. *Trametopsistasmanica* is characterised by its resupinate basidiomata, cream to pinkish-buff pore surface when fresh, becoming honey-yellow to snuff brown upon drying, cylindrical basidiospores (5.2–6.3 × 1.8–2.2 μm), and by growing on *Eucalyptus* sp. Detailed descriptions and illustrations of the two novel species are provided.

## ﻿Introduction

*Trametopsis* Tomšovský was established by Tomšovský (2008) with *T.cervina* (Schwein.) Tomšovský as type species. The morphological characteristics of *Trametopsis* are as follows: Basidiomata annual, sessile to effused-reflexed or rarely resupinate. Pileal surface pinkish buff to cinnamon or clay-buff, hirsute to strigose. Pore surface concolorous with pileal surface; pores irregular, daedaloid to irpicoid; dissepiments thin and lacerate. Context pale buff, fibrous. Tubes concolorous with the context, corky. Hyphal system dimitic; generative hyphae clamped. Cystidia absent; fusoid cystidioles occasionally present. Basidia clavate, bearing four sterigmata and a basal clamp connection. Basidiospores cylindrical, hyaline, thin-walled, smooth, IKI–, CB– (Tomšovský 2008).

Gómez-Montoya et al. (2017) evaluated the species of *Trametopsis* in the Neotropics based on phylogenetic evidences and morphological analyses. The phylogenetic analyses showed that *Trametopsis* is an independent genus; furthermore, one new species, *T.aborigena* Gómez-Mont. & Robledo, and the two new combinations, *T.brasiliensis* (Ryvarden & de Meijer) Gómez-Mont. & Robledo and *T.luteocontexta* (Ryvarden & de Meijer) Gómez-Mont., Robledo & Drechsler-Santos were presented. Westphalen et al. (2019) summarised *Antrodiella* Ryvarden & I. Johans. and related genera from the Neotropics, and *T.luteocontexta* was transferred to *Aegis* Gómez-Mont., Rajchenb. & Robledo according to morphological and molecular data. Recent phylogenetic studies have shown that *Trametopsis* belongs to Irpicaceae Spirin & Zmitr in the phlebioid clade (Justo et al. 2017; Chen et al. 2021). So far, three species are accepted in *Trametopsis*, viz., *T.aborigena*, *T.brasiliensis* and *T.cervina*.

During our investigations of wood-decay fungi, some specimens of the phlebioid clade were collected. These specimens possess glabrous or velutinate to strigose pileal surface, round to angular, irregular, daedaleoid to irpicoid pores, saprophytic on dead wood and causing white rot. Preliminary morphological observations showed that these specimens may belong to *Trametopsis*. To determine the phylogenetic positions of these specimens, we performed phylogenetic analyses of Irpicaceae with emphasis on *Trametopsis* based on the combined sequences datasets of ITS+nLSU and ITS+nLSU+RPB1+RPB2+TEF1. Combining morphological and molecular evidence, two new species, viz., *T.abieticola* and *T.tasmanica* are described and illustrated.

## ﻿Materials and methods

### ﻿Morphological studies

The examined specimens were deposited at the herbarium of the Institute of Microbiology, Beijing Forestry University (BJFC). Morphological descriptions and abbreviations used in this study follow Cui et al. (2019) and Song et al. (2021).

### ﻿Molecular studies and phylogenetic analysis

The procedures for DNA extraction and polymerase chain reaction (PCR) used in this study were the same as described by Liu et al. (2021a) and Sun et al. (2022). The ITS regions were amplified with the primer pairs ITS4 and ITS5, the nLSU regions were amplified with the primer pairs LR0R and LR7, RPB1 was ampliﬁed with primer pairs RPB1-Af and RPB1-Cr, RPB2 gene was amplified with the primer pairs fRPB2-f5F and bRPB2-7.1R, and TEF1 gene was amplified with the primer pairs EF1-983F and EF1-1567R (White et al. 1990; Rehner 2001; Matheny et al. 2002; Matheny 2005).

The PCR cycling schedules for different DNA sequences of ITS, nLSU, RPB1, RPB2 and TEF1 genes used in this study followed those used in Liu et al. (2021b, 2022) with some modifications. The PCR products were purified and sequenced at Beijing Genomics Institute, China, with the same primers. All newly generated sequences were submitted to GenBank and were listed in Table 1.

**Table 1.** A list of species, specimens, and GenBank accession number of sequences used for phylogenetic analyses in this study.

Species	Sample no.	Locality	GenBank accessions					References
			ITS	nLSU	RPB1	RPB2	TEF1	
*Byssomeruliuscorium*	FCUG 2701	Russia	MZ636931	GQ470630	MZ748415	OK136068	MZ913668	Wu et al. (2010); Chen et al. (2021)
*B.corium*	Wu 1207-55	China	MZ636932	MZ637096	—	—	—	Chen et al. (2021)
*B.corium*	FP-102382	USA	KP135007	KP135230	KP134802	KP134921	—	Floudas and Hibbett (2015)
*Ceriporiabubalinomarginata*	Dai 11327	China	JX623953	JX644045	—	—	—	Jia et al. (2014)
*C.bubalinomarginata*	Dai 12499	China	JX623954	JX644044	—	—	—	Jia et al. (2014)
*C.viridans*	Spirin 5909	Finland	KX236481	KX236481	—	—	—	Spirin et al. (2016)
*C.viridans*	Miettinen 11701	Netherlands	KX752600	KX752600	—	—	—	Miettinen et al. (2016)
Crystallicutiscf.serpens	Wu 1608-130	China	MZ636946	MZ637108	—	—	—	Chen et al. (2021)
C.cf.serpens	Wu 1608-81	China	MZ636947	MZ637109	MZ748435	OK136094	MZ913699	Chen et al. (2021)
*C.serpens*	HHB-15692	USA	KP135031	KP135200	KP134785	KP134914	—	Floudas and Hibbett (2015)
*C.* sp.	FP-101245	USA	KP135029	—	—	—	—	Floudas and Hibbett (2015)
*Cytidiellaalbida*	GB-1833	Spain	KY948748	KY948889	KY948960	OK136069	MZ913675	Justo et al. (2017); Chen et al. (2021)
*C.albomarginata*	Wei 18-474	China	MZ636948	MZ637110	MZ748429	OK136070	MZ913678	Chen et al. (2021)
*C.albomarginata*	Wu 0108-86	China	MZ636949	MZ637111	MZ748430	OK136071	MZ913677	Chen et al. (2021)
*C.albomellea*	FP-102339	USA	MZ636950	MZ637112	MZ748431	—	—	Chen et al. (2021)
*C.nitidula*	T-407	USA	KY948747	MZ637113	KY948961	OK136072	MZ913676	Justo et al. (2017); Chen et al. (2021)
*Efibulagracilis*	FD-455	USA	KP135027	MZ637116	KP134804	OK136077	MZ913679	Floudas and Hibbett (2015); Chen et al. (2021)
*E.gracilis*	FP-102052	USA	KP135028	—	—	—	—	Floudas and Hibbett (2015)
*E.matsuensis*	Wu 1011-18	China	MZ636956	MZ637119	MZ748418	OK136078	MZ913680	Chen et al. (2021)
*E.matsuensis*	Wu 1011-19	China	MZ636957	MZ637120	—	—	—	Chen et al. (2021)
*E.tropica*	Chen 3596	China	MZ636966	MZ637128	—	—	—	Chen et al. (2021)
*E.tropica*	Wei 18-149	China	MZ636967	MZ637129	MZ748419	OK136079	MZ913681	Chen et al. (2021)
*E.yunnanensis*	Wu 880515-1	China	MZ636977	GQ470672	MZ748420	OK136080	MZ913682	Wu et al. (2010); Chen et al. (2021)
*E.yunnanensis*	Wu 0910-104	China	MZ636976	MZ637138	—	—	—	Chen et al. (2021)
*Gloeoporusorientalis*	Wei 16-485	China	MZ636980	MZ637141	MZ748443	OK136095	MZ913709	Chen et al. (2021)
*G.pannocinctus*	L-15726	USA	KP135060	KP135214	KP134867	KP134973	—	Floudas and Hibbett (2015)
*Irpexflavus*	Wu 0705-1	China	MZ636988	MZ637149	MZ748432	OK136087	MZ913683	Chen et al. (2021)
*I.flavus*	Wu 0705-2	China	MZ636989	MZ637150	—	—	—	Chen et al. (2021)
*I.hacksungii*	F 2008	South Korea	FJ750851	—	—	—	—	Lee et al. (2008)
*I.hydnoides*	KUC 20121109-01	South Korea	KJ668510	KJ668362	—	—	—	Jang et al. (2016)
*I.laceratus*	WHC 1372	China	MZ636990	MZ637151	—	—	—	Chen et al. (2021)
*I.lacteus*	DO 421	Sweden	JX109852	JX109852	—	JX109882	—	Binder et al. (2013)
*I.lacteus*	FD-93	USA	KP135025	—	—	—	—	Floudas and Hibbett (2015)
*I.latemarginatus*	FP-55521-T	USA	KP135024	KP135202	KP134805	KP134915	—	Floudas and Hibbett (2015)
*I.latemarginatus*	Dai 7165	China	KY131834	KY131893	—	—	—	Wu et al. (2017)
*I.lenis*	Wu 1608-14	China	MZ636991	MZ637152	MZ748434	—	MZ913685	Chen et al. (2021)
*I.lenis*	Wu 1608-22	China	MZ636992	MZ637153	—	—	—	Chen et al. (2021)
*I.rosettiformis*	LR40855	USA	JN649347	JN649347	—	—	—	Sjökvist et al. (2012)
*I.rosettiformis*	Meijer3729	Brazil	JN649346	JN649346	—	JX109875	JX109904	Sjökvist et al. (2012); Binder et al. (2013)
*Leptoporusmollis*	LE BIN 3849	Russia	MG735341	—	—	—	—	Psurtseva (2010)
*L.mollis*	RLG-7163	USA	KY948794	MZ637155	KY948956	OK136101	MZ913693	Justo et al. (2017); Chen et al. (2021)
*Meruliopsisalbostramineus*	HHB 10729	USA	KP135051	KP135229	KP134787	—	—	Floudas and Hibbett (2015)
*M.crassitunicata*	CHWC 1506-46	China	LC427010	LC427034	—	—	—	Chen et al. (2020)
*M.leptocystidiata*	Wu 1708-43	China	LC427013	LC427033	LC427070	—	—	Chen et al. (2020)
*M.parvispora*	Wu 1209-58	China	LC427017	LC427039	LC427065	—	—	Chen et al. (2020)
*M.taxicola*	GC 1704-60	China	LC427028	LC427050	LC427063	—	—	Chen et al. (2020)
*Phanerochaetealbida*	GC 1407-14	China	MZ422788	MZ637179	MZ748384	OK136013	MZ913704	Chen et al. (2021)
*P.alnea*	FP-151125	USA	KP135177	MZ637181	MZ748385	OK136014	MZ913641	Floudas and Hibbett (2015); Chen et al. (2021)
*Phanerochaetellaangustocystidiata*	Wu 9606-39	China	MZ637020	GQ470638	MZ748422	OK136082	MZ913687	Wu et al. (2010); Chen et al. (2021)
*P.angustocystidiata*	GC 1501-20	China	MZ637017	MZ637225	—	—	—	Chen et al. (2021)
*P.exilis*	HHB-6988	USA	KP135001	KP135236	KP134799	KP134918	—	Floudas and Hibbett (2015)
*P.formosana*	Chen 479	China	MZ637023	GQ470650	MZ748424	OK136084	MZ913718	Wu et al. (2010); Chen et al. (2021)
*P.formosana*	Chen 3468	China	MZ637022	MZ637229	—	—	—	Chen et al. (2021)
*P.leptoderma*	Chen 1362	China	MZ637025	GQ470646	MZ748423	OK136083	MZ913689	Wu et al. (2010); Chen et al. (2021)
*P.leptoderma*	Wu 1703-9	China	MZ637027	MZ637232	—	—	—	Wu et al. (2010)
*P.xerophila*	HHB-8509	USA	KP134996	KP135259	KP134800	KP134919	MZ913688	Floudas and Hibbett (2015); Chen et al. (2021)
*P.xerophila*	KKN-172	USA	KP134997	—	—	—	—	Floudas and Hibbett (2015)
*Raduliporusaneirinus*	HHB-15629	USA	KP135023	KP135207	KP134795	—	—	Floudas and Hibbett (2015)
*R.aneirinus*	Wu 0409-199	China	MZ637068	MZ637267	—	OK136096	MZ913712	Chen et al. (2021)
*R.pseudogilvescens*	Wu 9508-54	China	MZ637069	MZ637269	—	—	—	Chen et al. (2021)
*Resiniporuspseudogilvescens*	Wu 1209-46	China	KY688203	MZ637268	MZ748436	OK136097	MZ913713	Chen et al. (2018); Chen et al. (2021)
*R.resinascens*	BRNM 710169	Czech Republic	FJ496675	FJ496698	—	—	—	Tomšovský et al. (2010)
***Trametopsisabieticola***	**Cui 18363**	**China**	**ON041038**	**ON041054**	**ON099403**	**ON099411**	**ON083777**	**Present study**
***T.abieticola***	**Cui 18383**	**China**	**ON041039**	**ON041055**	**ON099404**	**ON099412**	**ON083778**	**Present study**
*T.aborigena*	Robledo 1236	Argentina	KY655336	KY655338	—	—	—	Gómez-Montoya et al. (2017)
*T.aborigena*	Robledo 1238	Argentina	KY655337	KY655339	—	—	—	Gómez-Montoya et al. (2017)
*T.brasiliensis*	Meijer 3637	Brazil	JN710510	JN710510	—	—	—	Miettinena et al. (2012)
***T.cervina***	**Cui 17712**	**China**	**ON041040**	**ON041056**	—	**ON099413**	**ON083779**	**Present study**
***T.cervina***	**Cui 18017**	**China**	**ON041041**	**ON041057**	—	**ON099414**	**ON083780**	**Present study**
***T.cervina***	**Cui 18019**	**China**	**ON041042**	**ON041058**	**ON099405**	**ON099415**	**ON083781**	**Present study**
***T.cervina***	**Dai 21818**	**China**	**ON041043**	**ON041059**	**ON099406**	—	**ON083782**	**Present study**
***T.cervina***	**Dai 21820**	**China**	**ON041044**	**ON041060**	**ON099407**	**ON099416**	**ON083783**	**Present study**
***T.cervina***	**Dai 22804**	**China**	**ON041045**	**ON041061**	—	**ON099417**	**ON083784**	**Present study**
***T.cervina***	**Dai 23454**	**China**	**ON041046**	**ON041062**	—	—	**ON083785**	**Present study**
***T.cervina***	**He 6863**	**China**	**ON041047**	**ON041063**	**ON099408**	**ON099418**	**ON083786**	**Present study**
*T.cervina*	MG 299	Iran	KU213592	KU213594	—	—	—	—
*T.cervina*	TJV-93-216T	USA	JN165020	JN164796	JN164839	JN164877	JN164882	Justo and Hibbett (2011)
***T.tasmanica***	**Cui 16606**	**Australia**	**ON041048**	**ON041064**	**ON099409**	**ON099419**	**ON083787**	**Present study**
***T.tasmanica***	**Cui 16607**	**Australia**	**ON041049**	**ON041065**	**ON099410**	**ON099420**	**ON083788**	**Present study**

Newly generated sequences for this study are shown in bold.

Sequences were aligned with additional sequences downloaded from GenBank (Table 1) using ClustalX (Thompson et al. 1997). Alignment was manually adjusted to allow maximum alignment and to minimise gaps in BioEdit (Hall 1999). Sequence alignment was deposited to TreeBase (https://treebase.org/treebase-web; submission ID 29580). In phylogenetic reconstructions, the sequences of *Phanerochaetealbida* Sheng H. Wu and *P.alnea* (Fr.) P. Karst. obtained from GenBank were used as outgroups. The reason for choosing these two species as outgroup taxa is that they belong to *Phanerochaete* in Phanerochaetaceae, and are closely related to Irpicaceae (Chen et al. 2021), which conforms to the outgroup selection rules. Furthermore, species of *Phanerochaete* were also selected as outgroups in other phylogenetic studies of Irpicaceae, such as in El-Gharabawy et al (2021).

Phylogenetic analyses approaches used in this study followed Sun et al. (2020) and Ji et al. (2022). The congruencies of the 2-gene (ITS and nLSU) and 5-gene (ITS, nLSU, RPB1, RPB2 and TEF1) were evaluated with the incongruence length difference (ILD) test (Farris et al. 1994) implemented in PAUP* 4.0b10 (Swofford 2002), under heuristic search and 1000 homogeneity replicates. Maximum parsimony (MP) analysis was performed in PAUP* version 4.0b10 (Swofford 2002). Clade robustness was assessed using a bootstrap (BT) analysis with 1000 replicates (Felsenstein 1985). Descriptive tree statistics tree length (TL), consistency index (CI), retention index (RI), rescaled consistency index (RC), and homoplasy index (HI) were calculated for each Most Parsimonious Tree (MPT) generated. Maximum Likelihood (ML) analysis was performed in RAxML-HPC v. 8.2.3 with a GTR+G+I model (Stamatakis 2014). Bayesian inference (BI) was calculated by MrBayes 3.1.2 (Ronquist and Huelsenbeck 2003) with a general time reversible (GTR) model of DNA substitution and a gamma distribution rate variation across sites determined by MrModeltest 2.3 (Posada and Crandall 1998; Nylander 2008). The branch support was evaluated with a bootstrapping method of 1000 replicates (Hillis and Bull 1993).

Trees were viewed in FigTree v1.4.4 (http://tree.bio.ed.ac.uk/software/figtree/). Branches that received bootstrap supports for maximum parsimony (MP), maximum likelihood (ML) and Bayesian posterior probabilities (BPP) greater than or equal to 75% (MP and ML) and 0.95 (BPP) were considered as significantly supported, respectively.

## ﻿Results

### ﻿Phylogeny

**Figure 1.**
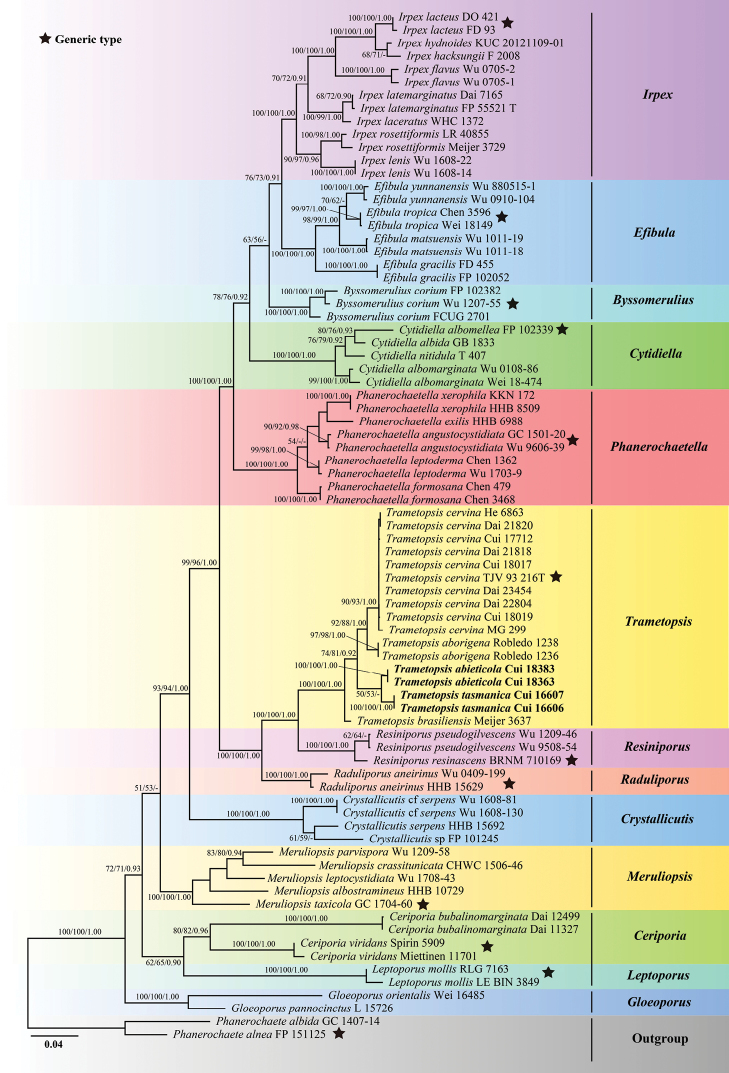
Maximum likelihood tree illustrating the phylogeny of *Trametopsis* based on the combined sequences dataset of ITS+nLSU. Branches are labelled with maximum likelihood bootstrap higher than 50%, parsimony bootstrap proportions higher than 50% and Bayesian posterior probabilities more than 0.90 respectively. Bold names = New species.

The combined 2-gene (ITS+nLSU) sequences dataset had an aligned length of 1893 characters, including gaps (619 characters for ITS, 1274 characters for nLSU), of which 1307 characters were constant, 105 were variable and parsimony-uninformative, and 481 were parsimony-informative. MP analysis yielded 26 equally parsimonious trees (TL = 2150, CI = 0.409, RI = 0.776, RC = 0.317, HI = 0.591). The best-fit evolutionary models applied in Bayesian analyses were selected by MrModeltest2 v. 2.3 for each region of the two genes, the model for ITS was GTR+I+G with equal frequency of nucleotides, while the model for nLSU was SYM+I+G with equal frequency of nucleotides. ML analysis resulted in a similar topology as MP and Bayesian analyses, and only the ML topology is shown in Fig. 1.

**Figure 2.**
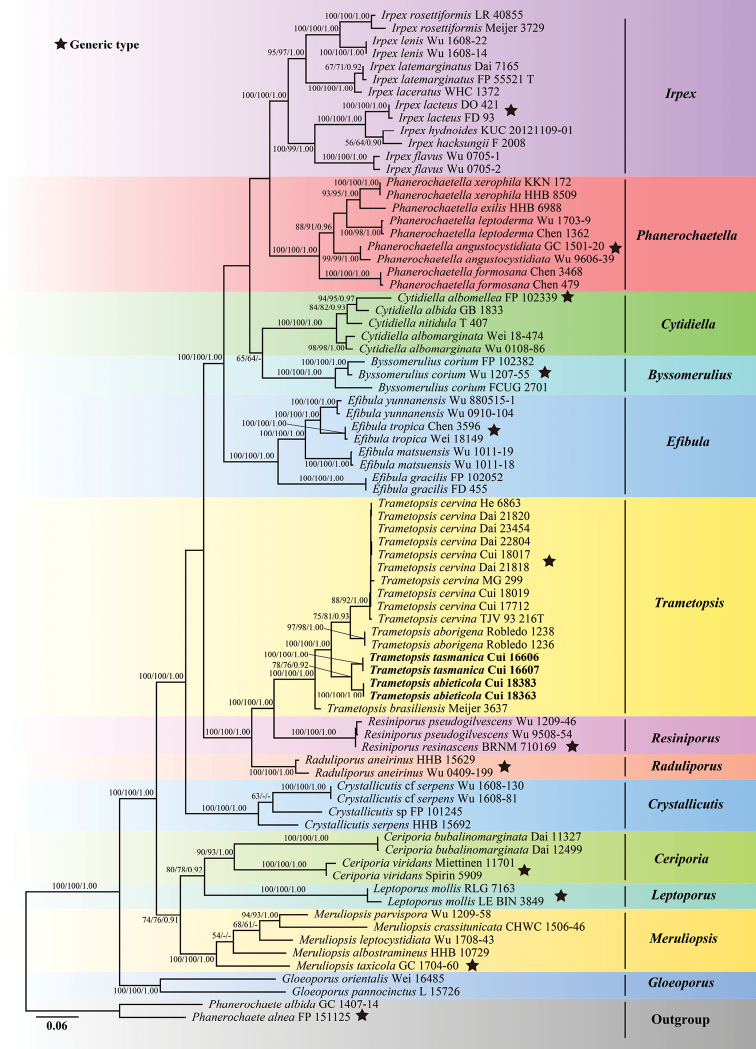
Maximum likelihood tree illustrating the phylogeny of *Trametopsis* based on the combined sequences dataset of ITS+nLSU+RPB1+RPB2+TEF1. Branches are labelled with maximum likelihood bootstrap higher than 50%, parsimony bootstrap proportions higher than 50% and Bayesian posterior probabilities more than 0.90 respectively. Bold names = New species.

The combined 5-gene (ITS+nLSU+RPB1+RPB2+TEF1) sequences dataset had an aligned length of 4609 characters, including gaps (619 characters for ITS, 1274 characters for nLSU, 1170 characters for RPB1, 1001 characters for RPB2, 545 characters for TEF1), of which 2675 characters were constant, 272 were variable and parsimony-uninformative, and 1662 were parsimony-informative. MP analysis yielded 36 equally parsimonious trees (TL = 9247, CI = 0.362, RI = 0.652, RC = 0.236, HI = 0.638). The best-fit evolutionary models applied in Bayesian analyses were selected by MrModeltest2 v. 2.3 for each region of the two genes, the model for ITS, RPB1, RPB2 and TEF1was GTR+I+G with equal frequency of nucleotides, while the model for nLSU was SYM+I+G with equal frequency of nucleotides. ML analysis resulted in a similar topology as MP and Bayesian analyses, and only the ML topology is shown in Fig. 2.

The phylogenetic trees inferred from ITS+nLSU and ITS+nLSU+RPB1+RPB2+TEF1 gene sequences were all obtained from 78 fungal samples representing 42 taxa of Irpicaceae and two taxa of Phanerochaetaceae within the phlebioid clade (Figs 1, 2). Phylogenetic analyses showed that *Trametopsisabieticola*, *T.aborigena*, *T.brasiliensis*, *T.cervina* and *T.tasmanica* grouped together within *Trametopsis* by high support (100% ML, 100% MP, 1.00 BPP; Figs 1, 2).

### ﻿Taxonomy

#### ﻿ Trametopsis abieticola

Taxon classificationFungiPolyporalesPolyporaceae

B.K. Cui & Shun Liu sp. nov.

0D3F936C-2A84-5213-A3B8-A9B7014DC52C

844097

Figs 3 , 4

##### Diagnosis.

*Trametopsisabieticola* is distinguished from *T.tasmanica* by larger pores (0.5–1 per mm) and basidiospores (5.8–7.2 × 1.9–2.6 μm), and by being distributed in the high altitude of mountains and growing on *Abies* sp.

##### Holotype.

China. Xizang Autonomous Region (Tibet), Mangkang County, Mangkang Mountain, on fallen trunk of *Abies* sp., 8 September 2020, Cui 18383 (holotype BJFC 035242).

##### Etymology.

*Abieticola* (Lat.): referring to the species grows on *Abies* sp.

##### Fruiting body.

Basidiomata annual, pileate, solitary or imbricate, soft corky to corky, without odour or taste when fresh, becoming corky and light in weight upon drying. Pilei applanate to flabelliform, projecting up to 9.5 cm long, 5.5 cm wide, and 2 cm thick at base. Pileal surface buff to buff-yellow when fresh, becoming pinkish buff to clay-buff when dry, strigose or glabrous; margin white to cream when fresh, becoming cream to buff-yellow when dry, obtuse to acute. Pore surface cream to buff when fresh, becoming pinkish buff to greyish brown upon drying; pores round to angular, 0.5–1 per mm; dissepiments slightly thick, entire to lacerate. Context corky, cream to buff yellow, up to 8 mm thick. Tubes concolorous with pore surface, corky, up to 7 mm long.

**Figure 3.**
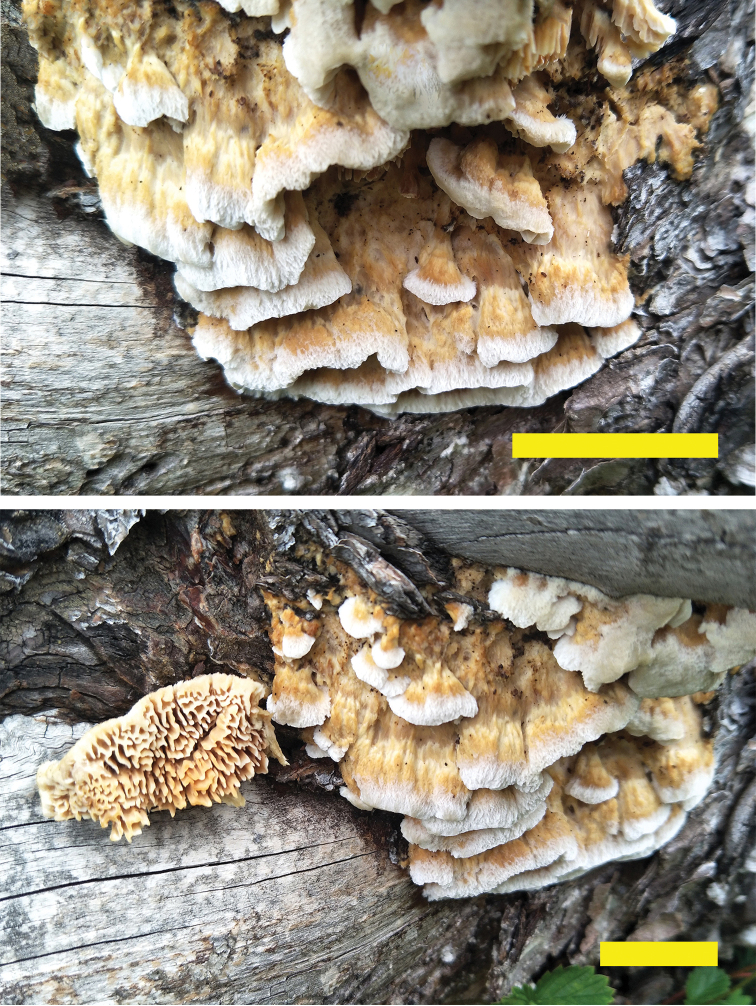
Basidiomata of *Trametopsisabieticola* (Holotype, Cui 18383). Scale bar: 3 cm.

##### Hyphal structure.

Hyphal system monomitic in context, dimitic in trama; generative hyphae with clamp connections; skeletal hyphae IKI–, CB–; tissues unchanged in KOH.

**Figure 4.**
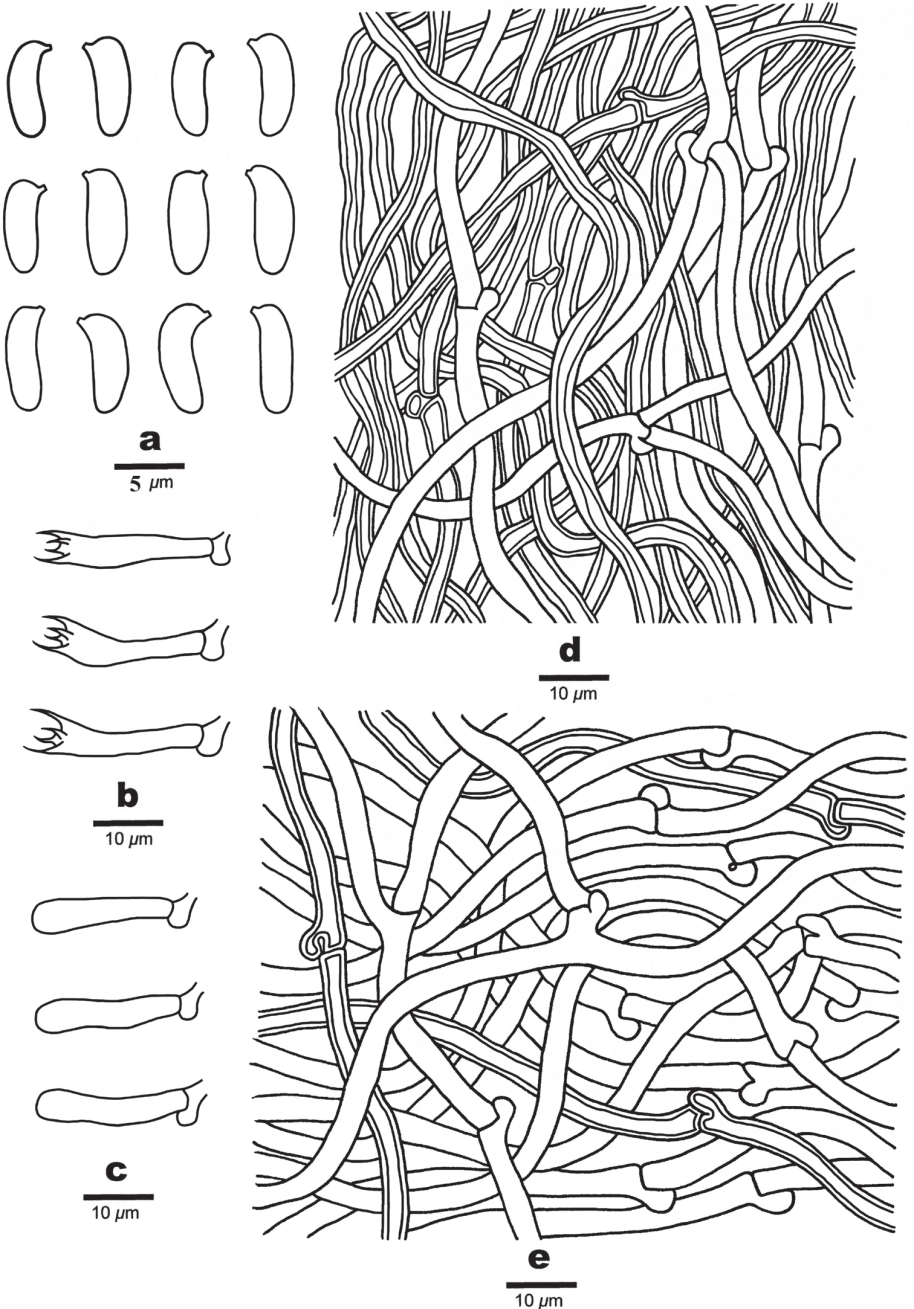
Microscopic structures of *Trametopsisabieticola* (Holotype, Cui 18383) **a** basidiospores **b** basidia **c** basidioles **d** hyphae from trama **e** hyphae from context.

##### Context.

Generative hyphae hyaline, thin- to slightly thick-walled, occasionally branched, loosely interwoven, 2.8–4.2 μm in diam.

##### Tubes.

Generative hyphae frequent, hyaline, thin- to slightly thick-walled, occasionally branched, 1.8–3.5 μm in diam.; skeletal hyphae dominant, hyaline, thick-walled with a wide to narrow lumen, occasionally branched, more or less straight, interwoven, 2–4.5 μm in diam. Cystidia and cystidioles absent. Basidia clavate, bearing four sterigmata and a basal clamp connection, 17.8–22.5 × 4.3–5.5 µm; basidioles dominant, similar to basidia but smaller.

##### Spores.

Basidiospores cylindrical, hyaline, thin-walled, smooth, IKI–, CB–, (5.7–)5.8–7.2 × (1.8–)1.9–2.6(–2.8) μm, L = 6.57 μm, W = 2.22 μm, Q = 2.75–3.26 (n = 60/2).

##### Type of rot.

White rot.

##### Additional specimen (paratype) examined.

China. Sichuan Province, Yajiang County, Kangbahanzi Village, on fallen trunk of *Abies* sp., 7 September 2020, Cui 18363 (BJFC 035222).

#### ﻿ Trametopsis tasmanica

Taxon classificationFungiPolyporalesPolyporaceae

B.K. Cui & Shun Liu sp. nov.

01D34EEF-3E37-59BB-972F-259196BA659F

844098

Figs 5 , 6

##### Diagnosis.

*Trametopsistasmanica* is distinguished from *T.abieticola* by resupinate basidiomata, smaller pores (2–4 per mm) and basidiospores (5.2–6.3 × 1.8–2.2 μm), and by growing on *Eucalyptus* sp.

##### Holotype.

Australia. Tasmania, Hobart, Mount Wellington, on rotten wood of *Eucalyptus* sp., 13 May 2018, Cui 16606 (holotype BJFC 029905).

##### Etymology.

*Tasmanica* (Lat.): referring to the species collected from Tasmania in Australia.

##### Fruiting body.

Basidiomata annual, resupinate, not easily separated from the substrate, without odour or taste when fresh, becoming corky to fragile and light in weight upon drying; up to 5.5 cm long, 2 cm wide, and 7 mm thick at centre. Pore surface cream to pinkish-buff when fresh, becoming honey-yellow to snuff brown upon drying; pores round to angular, 2–4 per mm; dissepiments slightly thick, entire to lacerate. Context very thin, corky, cream to buff, up to 2 mm thick. Tubes concolorous with pore surface, corky, up to 4 mm long.

**Figure 5.**
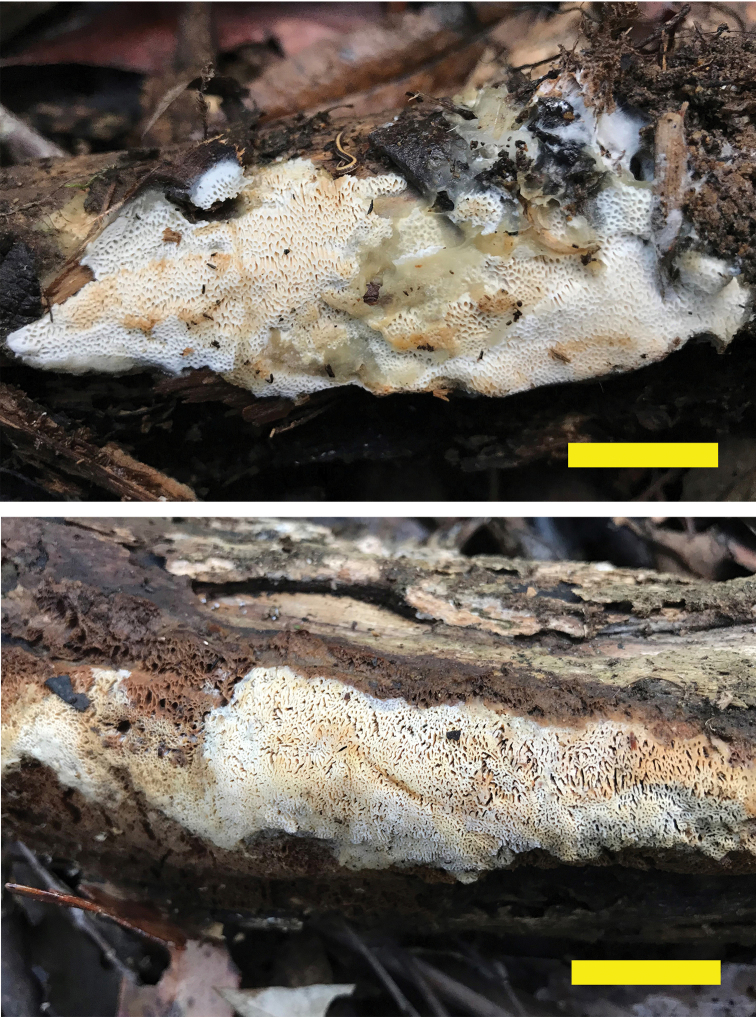
*Trametopsistasmanica* (Holotype, Cui 16606 and paratype, Cui 16607). Scale bar: 1 cm.

##### Hyphal structure.

Hyphal system monomitic in context, dimitic in trama; generative hyphae with clamp connections; skeletal hyphae IKI–, CB–; tissues unchanged in KOH.

##### Context.

Generative hyphae hyaline, thin- to slightly thick-walled with a wide lumen, occasionally branched, loosely interwoven, 2.7–4 μm in diam.

##### Tubes.

Generative hyphae frequent, hyaline, thin-walled, occasionally branched, 2–3 μm in diam.; skeletal hyphae dominant, hyaline, thick-walled with a wide to narrow lumen, occasionally branched, more or less straight, interwoven, 2–3.7 μm in diam. Cystidia and cystidioles absent. Basidia clavate, bearing four sterigmata and a basal clamp connection, 16–19.5 × 3.7–5 µm; basidioles dominant, similar to basidia but smaller.

**Figure 6.**
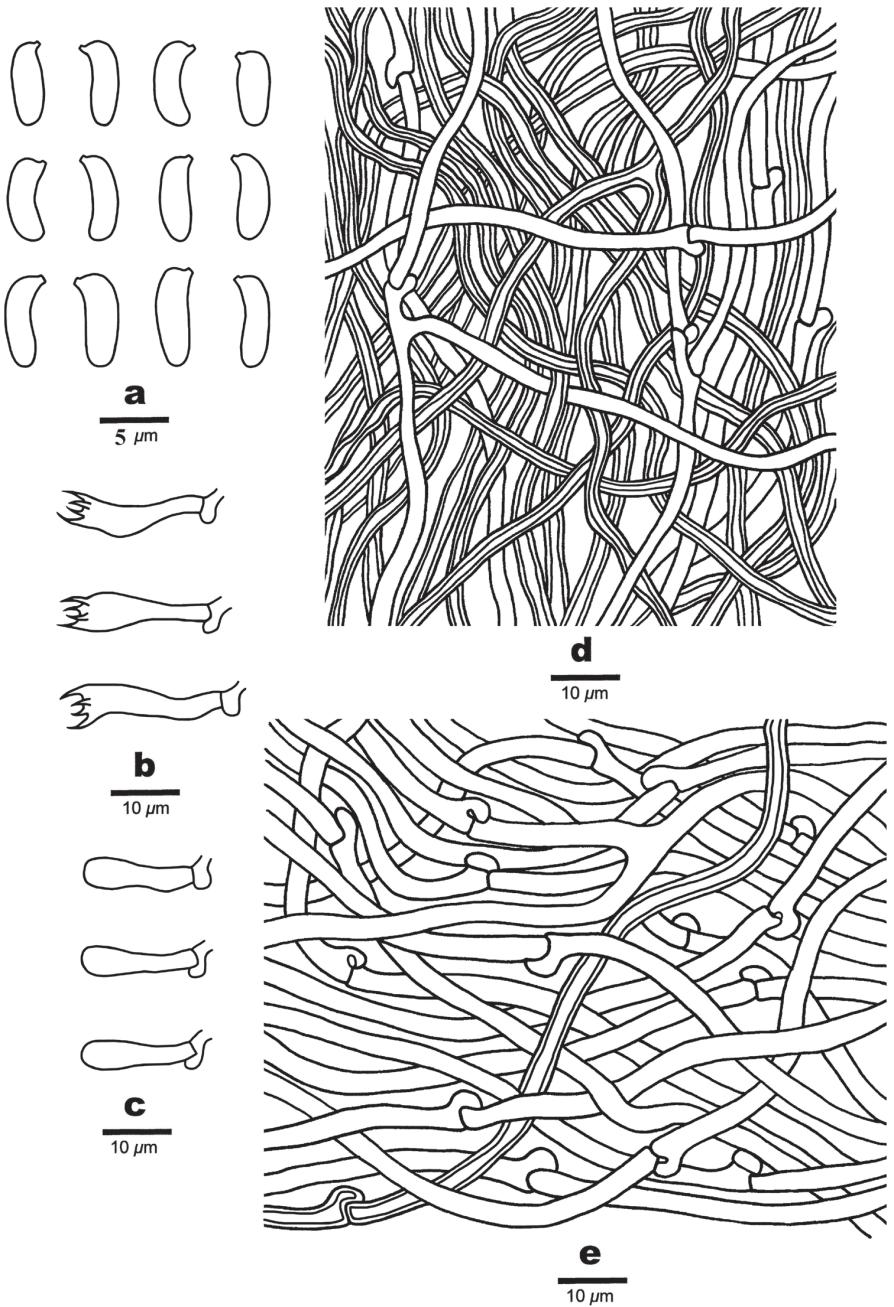
Microscopic structures of *Trametopsistasmanica* (Holotype, Cui 16606) **a** Basidiospores **b** Basidia **c** Basidioles **d** Hyphae from trama **e** Hyphae from context.

##### Spores.

Basidiospores cylindrical, hyaline, thin-walled, smooth, IKI–, CB–, (5–)5.2–6.3 × (1.7–)1.8–2.2(–2.4) μm, L = 5.84 μm, W = 2.02 μm, Q = 2.66–3.13 (n = 60/2).

##### Type of rot.

White rot.

##### Additional specimen (paratype) examined.

Australia. Tasmania, Hobart, Mount Wellington, on rotten branch of *Eucalyptus* sp., 13 May 2018, Cui 16607 (BJFC 029906).

## ﻿Discussion

**Figure 7.**
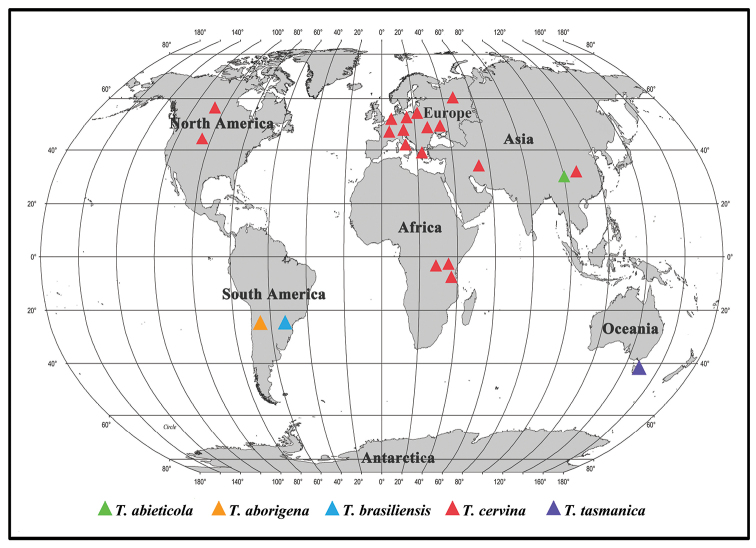
The geographical locations of the *Trametopsis* species distributed in the world.

In this study, the phylogenetic analyses of *Trametopsis* and related genera are inferred from the combined datasets of ITS+nLSU sequences (Fig. 1) and ITS+nLSU+RPB1+RPB2+TEF1 sequences (Fig. 2). The genera; *Raduliporus* Spirin & Zmitr., *Resiniporus* Zmitr. and *Trametopsis* grouped together and formed a highly supported lineage (Figs 1 and 2), which was called the *Trametopsis* lineage by Chen et al. (2021). Morphologically, *Raduliporus* and *Resiniporus* differ from *Trametopsis* by having a monomitic hyphal system and ellipsoid basidiospores (Chen et al. 2021). Phylogenetically, *T.abieticola* and *T.tasmanica* clustered with other *Trametopsis* species (Figs 1, 2) with high supports (100% MP, 100% ML, 1.00 BPP; Figs 1, 2). The main morphological characters and ecological habits of species in *Trametopsis* are provided in Table 2. The geographical locations of the *Trametopsis* species distributed in the world are indicated on the map (Fig. 7).

**Table 2.** The main morphological characters and ecological habits of species in *Trametopsis*. New species are shown in bold.

Species name	Distribution	Climate zone	Host	Fruiting body	Pores (per mm)	Basidia (μm)	Basidiospores (μm)	References
***Trametopsisabieticola***	**Asia (China)**	**Alpine plateau**	**Gymnosperm (*Abies*)**	**Pileate**	**0.5–1**	**17.8–22.5 × 4.3–5.5**	**5.8–7.2 × 1.9–2.6**	**Present study**
*T.aborigena*	South America (Argentina)	Neotropical	Angiosperm (Undetermined)	Pileate, effused-reflexed or occasionally resupinate	1–3	19–22 × 5–6	5–7 × 1–2.5	Gómez-Montoya et al. (2017)
*T.brasiliensis*	South America (Brazil)	Neotropical	Angiosperm (*Dicotyledonous*)	Pileate	1–2	15–20 × 4–5	4.5–5.5 × 1.8–2.2	Ryvarden and Meijer (2002); Gómez-Montoya et al. (2017)
*T.cervina*	Africa (Burundi, Rwanda, Tanzania), Asia (China, Iran), Europe (Austria, Belgium, Czech, France, Greece, Italy, Slovakia, Poland, Ukraine, Russia, etc.), and North America (Canada, USA)	Alpine plateau, temperate to tropical	Angiosperm (*Acer*, *Alnus*, *Betula*, *Carpinus*, *Elaeocarpus*, *Fagus*, *Juglans*, *Liquidambar*, *Populus*, *Quercus*, *Salix*, etc.); Gymnosperm (*Larix*, *Pinus*)	Effused-reflexed to pileate or occasionally resupinate	2–4	20–25 × 5–7	6–9 × 2–3	Tomšovský (2008); Gómez-Montoya et al. (2017); present study
***T.tasmanica***	**Oceania (Australia)**	**Temperate marine climate**	**Angiosperm (*Eucalyptus*)**	**Resupinate**	**2–4**	**16–19.5 × 3.7–5**	**5.2–6.3 × 1.8–2.2**	**Present study**

*Trametopsisabieticola* is distributed in high altitude areas of the Hengduan Mountains (altitude > 3500 m) and grows on *Abies* sp. In the phylogenetic trees, *T.abieticola* is closely related to *T.tasmanica* (Figs 1, 2). Morphologically, *T.tasmanica* differs from *T.abieticola* in having resupinate basidiomata, smaller pores (2–4 per mm) and basidiospores (5.2–6.3 × 1.8–2.2 μm), being distributed in Australia and growing on *Eucalyptus* sp. *Trametopsiscervina* can also distributed in high altitude areas of the Hengduan Mountains (according to our investigations), but *T.cervina* differs from *T.abieticola* by its smaller pores (2–4 per mm), longer basidiospores (6–9 × 2–3 μm; Tomšovský 2008), and usually growing on angiosperm trees. *Trametopsisaborigena*, *T.brasiliensis* and *T.abieticola* share an annual growth habit, a monomitic hyphal system in context, dimitic in trama and clamped generative hyphae; but *T.aborigena* differs from *T.abieticola* by having light pale brown to pale yellowish pileal surface with yellowish red to dark yellowish brown radial veins, smaller pores (1–3 per mm) and basidiospores (5–7 × 1–2 μm), and being distributed in neotropical regions of Argentina (Gómez-Montoya et al. 2017); *T.brasiliensis* differs from *T.abieticola* in having smaller pores (1–2 per mm) and basidiospores (4.5–5.5 × 1.8–2.2 μm), and being distributed in neotropical regions of Brazil (Gómez-Montoya et al. 2017).

*Trametopsistasmanica* is distributed in Tasmania, Australia and grows on *Eucalyptus* sp. Before that, there was no report of *Trametopsis* in Oceania. Morphologically, *T.tasmanica* and *T.cervina* share similar-sized pores, but *T.cervina* differs from *T.tasmanica* by its pileate to effused-reflexed basidiomata, larger basidiospores (6–9 × 2–3 μm; Tomšovský 2008). *Trametopsisaborigena*, *T.brasiliensis* and *T.tasmanica* are only distributed in the southern hemisphere and grow on angiosperm trees. However, *T.aborigena* differs from *T.tasmanica* by having pileate, effused-reflexed to occasionally resupinate basidiomata, larger basidia (19–22 × 5–6 μm) and basidiospores (5–7 × 1–2.5 μm), and being distributed in neotropical regions of Argentina (Gómez-Montoya et al. 2017); *T.brasiliensis* differs from *T.tasmanica* in having pileate basidiomata, larger pores (1–2 per mm) and distributed in neotropical regions of Brazil (Gómez-Montoya et al. 2017).

In summary, we performed a taxonomic and phylogenetic study of *Trametopsis*. The concepts and species number of the *Trametopsis* are updated. So far, five species are accepted in the *Trametopsis* around the world. Currently, *Trametopsis* is characterised by an annual growth habit, effused-reflexed to pileate or resupinate, solitary or imbricate basidiomata, pinkish buff to cinnamon or clay-buff, zonate or azonate, glabrous or velutinate to strigose pileal surface, cream, pale yellow to greyish brown pore surface with round to angular, irregular, daedaleoid to irpicoid pores, a monomitic hyphal system in context, dimitic in trama, clamped generative hyphae, and allantoid to cylindrical basidiospores; it grows on different angiosperm and gymnosperm trees, causing white rot of wood (Tomšovský 2008; Gómez-Montoya et al. 2017).

## Supplementary Material

XML Treatment for Trametopsis abieticola

XML Treatment for Trametopsis tasmanica
